# Improvement of photon extraction efficiency of GaN-based LED using micro and nano complex polymer structures

**DOI:** 10.1186/1556-276X-6-578

**Published:** 2011-10-31

**Authors:** Joong-Yeon Cho, Kyeong-Jae Byeon, Hyoungwon Park, Jinseung Kim, Hyeong-Seok Kim, Heon Lee

**Affiliations:** 1 Department of Materials Science and Engineering, Korea University, Anam-dong 5-ga, Seongbuk-gu, Seoul 136-713, South Korea; 2 Department of Electrical and Electronics Engineering, Chung-Ang University, Seoul 156-756, South Korea

## Abstract

A micro- and nanoscale complex structure made of a high refractive index polymer (*n *= 2.08) was formed on the ITO electrode layer of an edge-emitting type GaN blue light-emitting diode (LED), in order to improve the photon extraction efficiency by suppressing total internal reflection of photons. The nanoimprint lithography process was used to form the micro- and nanoscale complex structures, using a polymer resin with dispersed TiO_2 _nano-particles as an imprint resin. Plasma processing, such as reactive ion etching, was used to form the micro- and nano-scale complex structure; thus, plasma-induced damage to the LED device can be avoided. Due to the high refractive index polymeric micro- and nanostructure on the ITO layer, the electroluminescence emission was increased up to 20%, compared to an identical LED that was grown on a patterned sapphire substrate to improve photon extraction efficiency.

## Introduction

High brightness GaN-based light-emitting diodes (LEDs) have been widely used for solid-state lighting sources due to their low power consumption, long lifetime, compact form factor, and eco-friendly nature [1-3]. The internal quantum efficiency (*η*_int_) of GaN-based LEDs has been drastically improved by the progress of GaN-based epitaxial growth and device fabrication technologies [4,5]. Accordingly, many attempts have been made to maximize the external quantum efficiency (photon extraction efficiency) of LEDs. However, much room remains for improvement of the external quantum efficiency.

One of the biggest issues surrounding the current high brightness LEDs is their low light extraction efficiency (*η*_ext_) due to the total internal reflection (TIR) of light at the interface of the LED structure with ambient [6]. Various attempts, including surface roughening [7,8], the formation of photonic crystals [9,10], the use of patterned sapphire substrates (PSS) [11,12], and the use of an air-gap structure inside the LED [13], were made to suppress the TIR.

The TIR can be minimized by the scattering of light at the interface, which was enhanced by forming the photonic crystal structure or other micro- and nanoscale complex structures. In order to form those structures, plasma processing, such as reactive ion etching (RIE) or inductive coupled plasma etching, is inevitably used along with the lithography process and this can deteriorate the LED's electrical performance [14-16]. Therefore, micro- or nanoscale complex structures need to be formed on the LED structure without plasma processing.

In this study, micro- and nanoscale complex structures made of high refractive index polymer were formed to enhance the LED light extraction efficiency. The micro- and nanoscale structures were obtained from the photo-electro chemical (PEC)-etched surface of the N-faced GaN. The GaN epilayer of a vertical LED was detached from the sapphire substrate and placed over metallic heat sink; thus, the N-faced GaN surface was exposed. In order to improve the photon extraction efficiency of the vertical LED, the N-faced GaN surface was etched using the PEC process to form micro- and nanoscale structure [17]. Micro- and nanoscale patterns of N-faced GaN was replicated using a polydimethylsiloxane (PDMS) molding process and transferred to the ITO electrode surface of conventional edge emitting type GaN blue LED devices using nanoimprint lithography. Due to the micro- and nanoscale complex structures that had formed on the ITO layer, the TIR could be suppressed and more photons could be extracted by scattering with the structure.

## Experimental procedure

### Fabrication of micro and nano complex structures on the GaN-based LED

**Figure 1:**
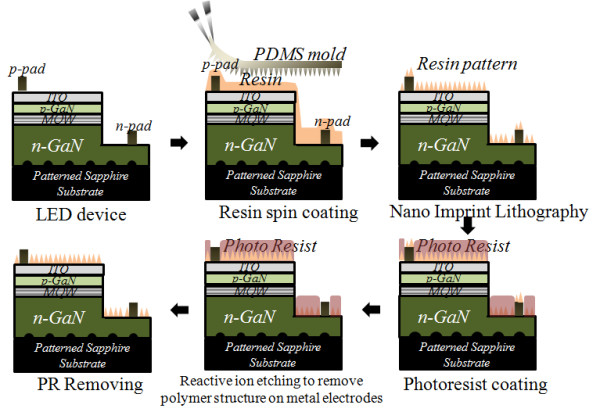
**Schematic diagram of fabrication of micro- and nanoscale complex polymer structure on the LED device**.

The overall process flow used to form the polymeric micro and nano complex structure on LED device is described in Figure 1. Details of the fabrication of the LED devices have been shown elsewhere [18]. As shown in Figure 1, a micro and nano complex polymer structure was formed on the completed LED structure, which has n and p electrodes and a mesa structure using the nanoimprint lithography process. Since a flexible PDMS stamp was used as an imprint template, a micro- and nanoscale complex polymer structure was uniformly formed on the LED despite the step height between the n- and p-GaN regions. The process details of PDMS replication are available elsewhere [19]. The polymer structure on the electrodes of the p- and n-GaN layers was selectively removed by photolithography and RIE. GXR601, which purchased from AZ Electronic Materials (Stockley Park, UK), was used as a positive photo-resist. Since only metal electrodes were exposed and the ITO surface was not exposed to the plasma, plasma-induced damage to the LED device was avoided.

### Fabrication of mico- and nanoscale complex structure using PEC etching process

The complicated micro- and nanoscale structure originated from the photochemical etched N-face GaN epitaxial layer. The GaN layer was epitaxially grown on (0001) sapphire substrate with a thickness of a few micrometers and was lifted off using a laser. The N-faced GaN surface was then exposed and etched with 5 M KOH solution at 60°C. To enhance the etching, the solution was continuously stirred and ultraviolet light was illuminated simultaneously onto the surface during the etching process [17].

### Details of material used as micro- and nanoscaled complex structure

A high refractive index resin containing TiO_2 _nanoparticles was purchased from Brewer Science Inc. (Rolla, MO, USA) and used as an imprint resin to form the micro- and nanoscale complex structures since its *n *and *k *values are 2.08 and 0.004, respectively, at 450 nm, the blue LED emission wavelength. The transmittance of the high refractive index is > 90% at the blue LED emission wavelength [20].

### Analysis of the morphologies and the property of the LED

The morphologies of micro- and nanoscale complex structures of the high refractive index polymer were analyzed by scanning electron microscopy (SEM) and atomic force microscopy (AFM). The effect of the micro- and nanoscale complex structure on the enhancement of the LED light extraction efficiency was confirmed by electroluminescence measurement. The electrical properties of the LED devices were measured using current-voltage (I-V) characteristics.

## Results and discussion

### Fabrication sub-micron polymer structure on the LEDs

**Figure 2:**
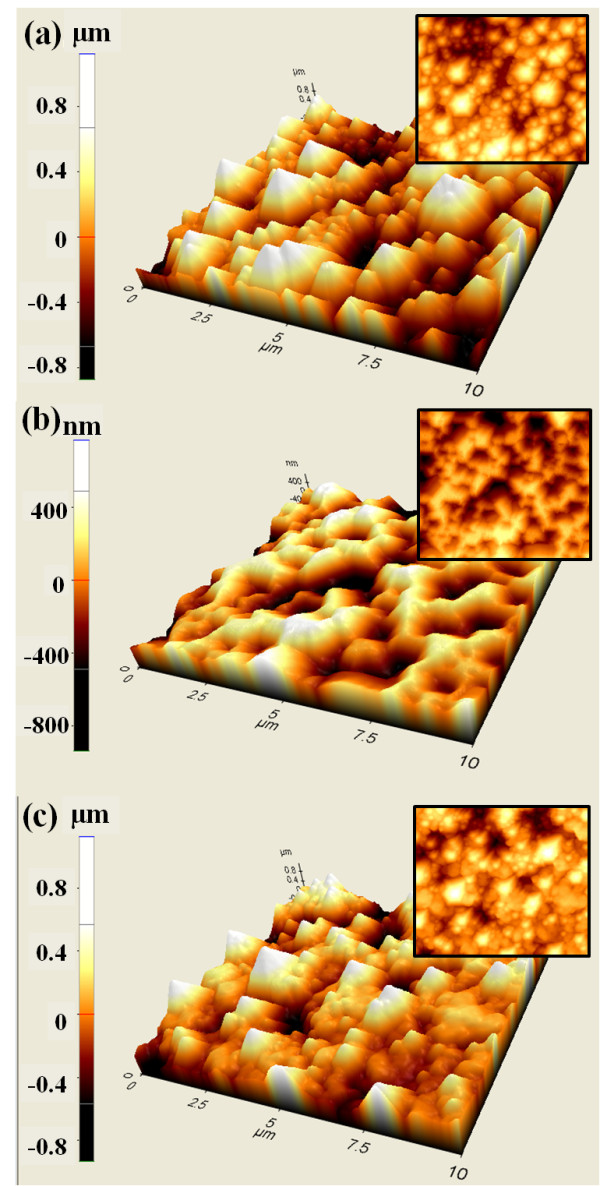
**Three-dimensional atomic force microscopy image (inset is a 2 dimensional image) of a micro- and nanoscale complex structure of (**a**) the N-faced GaN surface, (**b**) replicated polydimethylsiloxane stamp, and (**c**) surface of light-emitting diode device after the nanoimprint lithography process**.

The AFM analysis was performed to determine the morphology and height of the micro- and nanoscale complex structure. The AFM images of the micro- and nanoscale complex structure that formed on the N-face n-GaN surface, replicated polymer mold, and LED device are shown is Figure 2a,c, respectively. Figure 2 clearly shows that a continuous array of micro- and nanoscale structures was formed on the LED devices with high fidelity. According to AFM analysis, the height of the micro- and nanoscale complex polymer pattern ranged from 0.18 to 1.2 μm.

**Figure 3:**
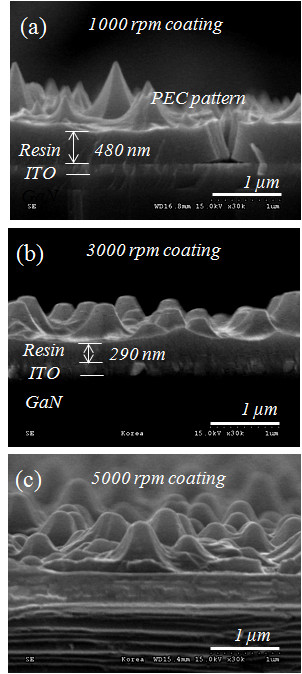
**Cross-sectional SEM micrograph of micro and nano-complex polymer structure formed on LED device with spin coating speed of (**a**) 1,000 rpm, (b) 3,000 rpm, and (**c**) 5,000 rpm**.

Since the actual LED device has a mesa structure, controlling the residual layer was extremely difficult. The residual polymer layer can have a detrimental effect on the transparency of the ITO layer of the GaN LED, so the spin-coating speed of the high refractive index polymer resin was carefully adjusted. As shown in Figure 3a,c, in case of lower spin-coating speeds, high fidelity pattern transfer was achieved and the thickness of the residual layer was also quite high. In cases of higher spin-coating speed, the micro- and nanoscale complex structure was not completely transferred due to the lack of an imprint resin. However, the thickness of the residual layer was drastically decreased. In this study, residual layer thickness control was accomplished by spin-coating speed adjustment rather than by the RIE. With a spin-coating speed of 5,000 rpm, micro- and nanoscale pattern transfer was achieved with high fidelity and the residual layer thickness was minimized.

**Figure 4:**
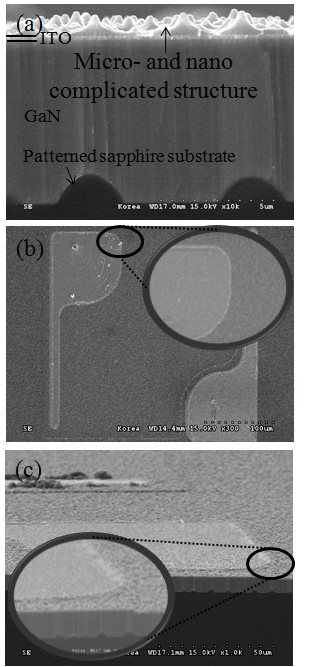
**Scanning electron microscopy (SEM) image of (a) cross-sectional view of the light emitting diode (LED) that was grown on the patterned sapphire substrate; SEM image of surface of the LED device after the reactive ion etching process in the (**b**) top view and (**c**) tilt view**.

In order to investigate the effect of the micro- and nanoscale polymer structures on the LED photon extraction efficiency, we chose two identical GaN-based blue LED devices that were grown on PSS and formed the micro- and nanoscale complex polymer structure on one wafer using the nanoimprint lithography process. The cross-sectional SEM micrographs of the LED structure with the micro- and nanoscale complex polymer structure on the PSS are shown in Figure 4a. Figure 4b, c show that the micro- and nanoscale complex polymer structures formed on the metal electrode were clearly removed via the RIE etching process to ensure stable current injection.

### Analysis of properties of the LEDs

**Figure 5:**
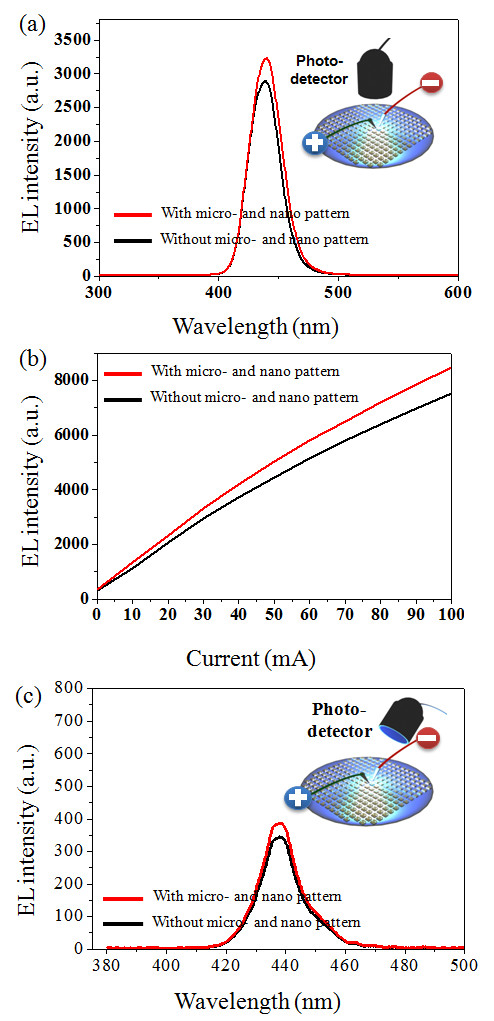
**The optical power of the electroluminescence emission of a light emitting diode that was grown on the patterned sapphire substrate with or without micro- and nanoscale complex structures: (a) with respect to wavelength at 20 mA, (b) with respect to current, and (c) with a 60° tilt**.

We measured the electroluminescence (EL) intensities at 20 mA of injection current LED devices with and without micro- and nanoscale complex polymer structures. PSS were used for both LED devices. The EL measurement was taken from one randomly selected device, measured ten times, and then averaged. As shown in Figure 5a, the EL emission of the LED structure with micro- and nanoscale complex polymer structures increased up to 13%. This increase in photon extraction efficiency is additional; thus, it is very meaningful since the LED structure was grown on a PSS wafer to increase the photon extraction efficiency up to 30% [11,12]. The microscale surface protrusion pattern on the PSS wafer already compensated the light direction to make it fit inside the escape cone, thus significant light extraction efficiency enhancement of the device was reported. Micro- and nanoscale complex polymer structures allow the photons to be extracted out of the LED structure via the photon scattering effect. In addition, the EL was measured in the other direction (angle) at a 60° tilt as shown in Figure 5c. In this case, similar EL emission intensity enhancement was observed compared with normal EL measurement.

**Figure 6:**
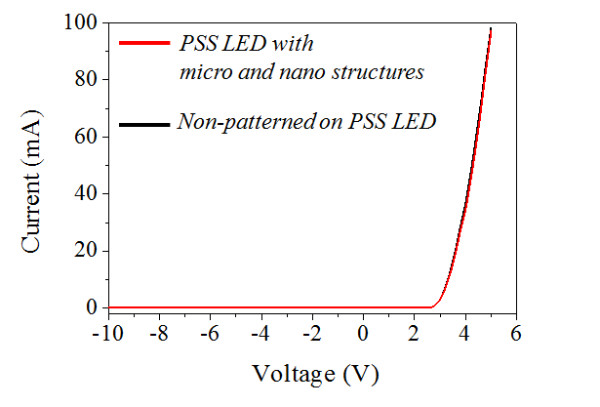
**I-V characteristics of the patterned and non-patterned section of the LED device**.

In order to confirm the effect of the nanoimprint patterning process on the electrical performance of the LED devices, I-V measurements were performed for the LED devices, including those with the micro- and nanoscale complex structures. In all cases, identical I-V characteristics were observed and the turn-on voltage and leakage current levels of the LED devices remained unchanged as shown in Figure 6. This finding implies that no electrical degradation was induced by the patterning process.

## Conclusions

The micro- and nanoscale complex structures were formed on the LED devices using the nanoimprint process. A high refractive index resin containing the TiO_2 _nanoparticles was used as the imprint resin. The I-V characteristics showed that the electrical performance of the LED devices was not degraded by the process used to fabricate the micro- and nanoscale structures. The EL intensity of the LED devices was increased by up to 13% for the LED structures grown on the PSS.

## Competing interests

The authors declare that they have no competing interests.

## Authors' contributions

CJY carried out overall experiments including nanoimprint lithography works as the first author. KJB carried out the fabrication of mico- and nanoscale complex strcutre using PEC etching process. HP was in charge of replication of Si mold using PDMS molding process. JK carried out the fabrication of the LED devices. HSK was in charge of the analysis of property of the LED devices. HL conducted design and analysis of all experiments as a corresponding author. All authors read and approved the final manuscript.
